# Revised management of advanced primary colon cancer: Case series of 2 patients

**DOI:** 10.1016/j.ijscr.2019.01.046

**Published:** 2019-02-10

**Authors:** Paul H. Sugarbaker, Mohamed T. Hassanein

**Affiliations:** a Center for Gastrointestinal Malignancies, Program in Peritoneal Surface Oncology, Washington Cancer Institute, Washington, DC, USA; b Department of Surgery, MedStar Washington Hospital Center, Washington, DC, USA

**Keywords:** Peritoneal metastases, Local recurrence, Peritonectomy, Hyperthermic intraperitoneal chemotherapy, HIPEC, Early postoperative intraperitoneal chemotherapy, EPIC, Colonoscopy, CT, Case series

## Abstract

**Background:**

The resection of a primary colon cancer has the opportunity to cure the patient of cancer by complete clearance and absolute containment of disease. Alternatively, if the cancer resection does not provide clearance and containment it will eventuate in local recurrence and peritoneal dissemination.

**Methods:**

Culpability of recurrence of colorectal cancer is difficult to determine. Are the management strategies of the primary cancer evaluation and treatment at fault or is the underlying disease process is to be held responsible? The clinical and radiologic findings that could have been evaluated by a multidisciplinary team (MDT) were critically evaluated in 2 patients with right colon adenocarcinoma. Strategies to accomplish complete clearance and absolute containment of the primary malignancy as resectable for cure were suggested.

**Results:**

Clinical evaluation of these 2 patients suggested that more knowledgeable preoperative evaluation by the multidisciplinary team (MDT) should have placed them in a high risk group for local recurrence and/or peritoneal dissemination. High carcinoembryonic antigen (CEA) tumor marker and by CT a large primary cancer infiltrating adjacent structures can be used to select advanced pre- and intraoperative treatment strategies.

**Conclusions:**

Two patients who had an approximately 50% possibility of long-term survival with optimal preoperative evaluation and expert surgical resection techniques may have been converted to a greatly reduced survival because of tumor dissemination and positive margins of resection at the time of primary cancer resection. Neither patient was evaluated by an MDT preoperatively. It is possible that these two patients entered the operating room with a contained malignancy but as result of suboptimal pre- and intraoperative management left the operating room with disseminated intraperitoneal disease.

## Highlights

• The surgical removal of a primary colon cancer demands complete clearance and absolute containment of the malignant process.
• Not all colon cancers are the same although the resection strategy varies little or not at all with the clinical features of the disease.
• The patient may enter the operating theater with a contained malignant process and leave with disseminated disease.
• Clinical and radiologic features of colon cancer indicate a high risk for cancer dissemination as a result of the resection.
• The surgeon must document in the medical record preoperatively the likelihood of clearance and containment.

## 1 Introduction

In 1967 Turnbull and colleagues presented at the American Surgical Society Meeting a document that has become part of the history of colorectal cancer surgery [1]. They advocated two features of primary colon cancer resection. Gentle handling of the segment of bowel containing the primary cancer and high ligation of lymphovascular pedicle to this part of the colon. They suggested that “no-touch isolation technique” produced a 5-year survival of 51% as compared to survival with conventional technique of 35%. The new approach was credited with reduced traumatic disruption of cancer into the portal blood and therefore fewer liver metastases. Enker et al. in 1979 agree with Turnbull et al. that a wide resection of the primary cancer and gentle manipulation was crucial, but credited the extended lymph node dissection that must occur with high lymphovascular ligation with improved survival [2]. I reviewed in 1982, 93 manuscripts to critically evaluate possible benefits of different resection techniques for both colon and rectal cancer [3]. My conclusion was that en bloc removal of the primary cancer with negative margins and an absence of surgical trauma that may result in local spillage of cancer cells was the most important feature of an optimal resection technique. In 1986, Quirke and colleagues concluded that lateral spread of rectal cancer to the margin of resection resulted in pelvic recurrence in 12 of 14 rectal cancer patients [4]. These data led to the call for a “total mesorectal excision” of rectal cancer to resect in the absence of positive margins and specimen trauma to avoid contamination of the pelvis with cancer cells [5]. With these concepts in mind, preoperative evaluation of rectal cancer, neoadjuvant treatments and total mesorectal excision are now a standard of care for rectal cancer.

Fixed requirements for preoperative evaluation and optimal resection techniques do not exist for colon cancer. In this opinion paper I ask that all patients with colon cancer be subjected to a review by the multidisciplinary team (MDT) to avoid as is possible narrow or positive margins of resection and tumor spillage from trauma to the primary cancer. The clinical course of two patients are presented to identify preoperative selection of special treatments and intraoperative changes in resection techniques that may improve outcomes.

Data on these 2 patients was prospectively recorded and then retrospectively reviewed at an academic institution. This research work has been reported in line with the PROCESS criteria [6]. This study was registered as a case series on the www.researchregistry.com website with UIN 4417.

## 2 Patient presentations

### 2.1 Patient 1

**Fig. 1:**
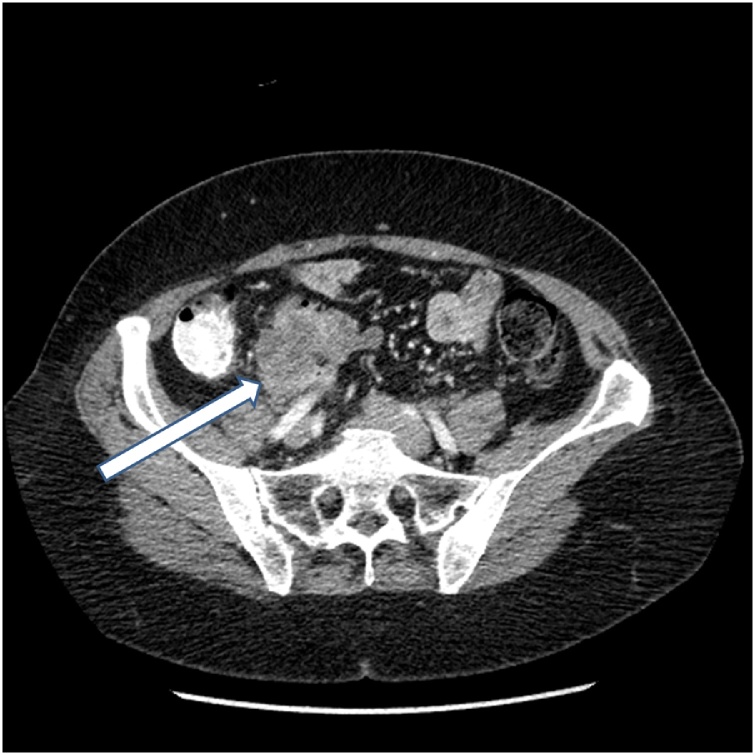
CT slice through a primary caecal adenocarcinoma. The greatest dimension of the mass was 8 cm. The arrow indicates the cancer extension into the retroperitoneum. Colonoscopic biopsy showed a poorly differentiated cancer.

In November of 2016 a 56 year old woman received the diagnosis of a caecal adenocarcinoma at the time of a screening colonoscopy. The carcinoembryonic antigen (CEA) blood test was 90. A CT was performed in December of 2016 and a single slice through the cancer is shown in Fig. 1. The mass measured 8 cm in greatest diameter. Its posterior borders suggested local penetration into adjacent retroperitoneal tissues. Lymph node involvement was suspect with a node greater than 1.5 cm in diameter.

In December of 2016 the patient was taken to the operating theater. By laparoscopic exploration no peritoneal or ovarian metastases were seen. The mass was mobilized by laparoscopic dissection and the proximal small bowel and ascending colon were divided without incident. The dissection posteriorly was difficult. To avoid damage to the right ureter a right ureteral stent was placed and the laparoscopic resection was converted to open [7].

The pathology report showed a poorly differentiated T_3_N_2_M_0_ malignancy. The margins of resection were not involved by cancer. The patient recovered from the surgery without incident. CEA decreased to normal range.

Because of the positive lymph nodes, the patient was recommended for adjuvant chemotherapy with 5-fluorouracil and oxaliplatin (FOLFOX regimen) [8]. In December of 2017, an increasing CEA blood test to 20 ng/ml was noted and a follow-up CT obtained. A mass was identified in the left mid-abdomen and was biopsied under CT guidance. Pathology showed adenocarcinoma that was histologically the same as the primary caecal malignancy. A full course of 5-fluorouracil with irinotecan (FOLFIRI) was administered [9]. On systemic chemotherapy the patient remained asymptomatic but the CEA increased to 74 ng/ml.

**Fig. 2:**
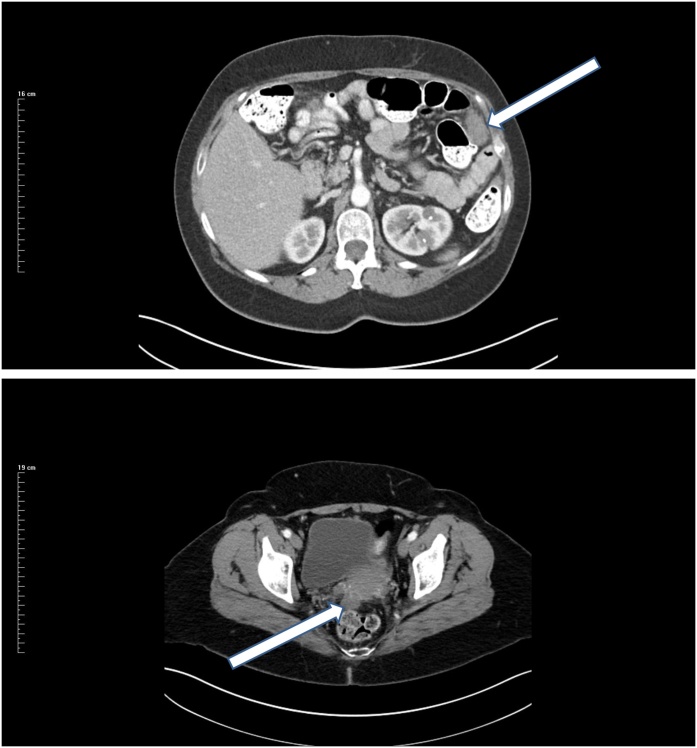
(Top) CT slice through the mid-abdomen at one year follow-up shows tumor nodules thought to be located in the greater omentum. (Bottom) CT slice through the pelvis shows a 4 cm mass in the rectouterine (cul de sac) space.

In May of 2018, the patient was evaluated for cytoreductive surgery (CRS) and possible hyperthermic intraperitoneal chemotherapy (HIPEC) [10]. CT showed masses in the omentum and rectouterine space (Fig. 2). In May of 2018, the patient underwent an exploratory laparotomy. There was a large volume of adenocarcinoma at the right colon resection site. There were multiple cancer nodules along the course of the right ureter. Four nodules approximately 3 cm in diameter were present within the greater omentum. A solitary nodule 4 cm in diameter was present in the rectouterine space. At the time of an 8-hour cytoreductive surgical procedure all these sites of recurrent disease were resected and determined to be infiltrated by adenocarcinoma compatible with the primary cancer specimen. The patient received HIPEC with melphalan and early postoperative intraperitoneal pegylated liposomal doxorubicin (Doxil) [11]. The peritoneal cancer index was 16 and the completeness of cytoreduction score was 0 [12]. Postoperatively, the patient developed absolute neutropenia on postoperative days 5 through 8 requiring treatment with filgrastim (Neupogen®). Postoperatively, her CEA blood test returned to normal and she is currently on surveillance by CEA blood tests and CT.

### 2.2 Patient 2

**Fig. 3:**
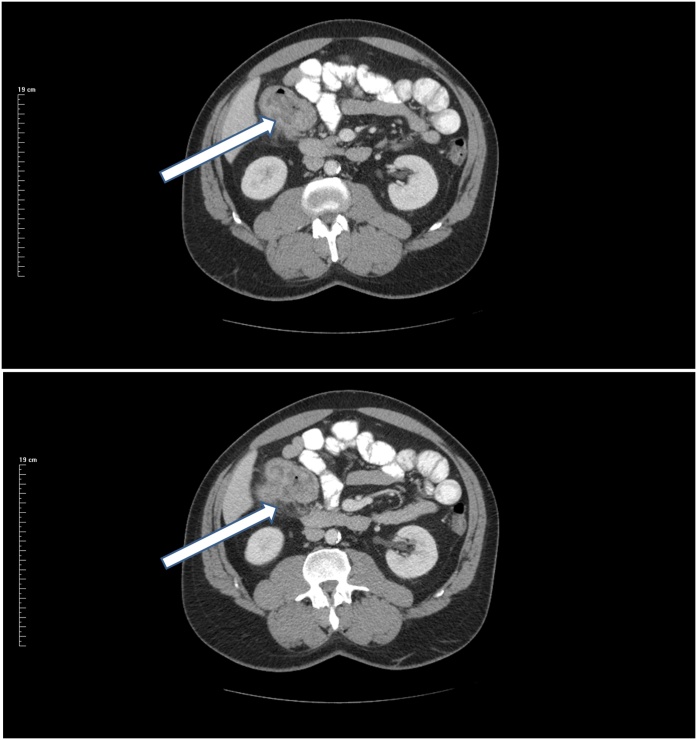
(Top) CT slice through a primary ascending colon adenocarcinoma. The greatest dimension of the mass was 8 cm. The mass touches the visceral surface of the right lobe of the liver and the second portion of the duodenum. (Bottom) CT slice shows stranding of the fat posteriorly suggesting a localized perforation.

A 55 year old man was diagnosed with anemia by his family physician towards the end of 2017. In February of 2018, an upper GI endoscopy and colonoscopy were performed which showed a right colon cancer. CEA was 40.4. CT of the chest, abdomen, and pelvis was obtained in February of 2018. This showed a mass 7 cm in greatest diameter that was immediately adjacent to the undersurface of the right liver and contiguous with numerous loops small bowel and the lowest part of the second portion of the duodenum (Fig. 3). There were mildly prominent mesenteric lymph nodes but no evidence of distant metastatic disease.

In February of 2018, the patient underwent a single port laparoscopic right colon resection. The surgeon described the tumor as stuck to the undersurface of the liver with adhesions taken down without difficulty. On histopathologic examination, the tumor was PT3N0M0 with 0/16 positive nodes. Perineural invasion was identified. The cancer was moderately to poorly differentiated. Systemic chemotherapy was not recommended.

**Fig. 4:**
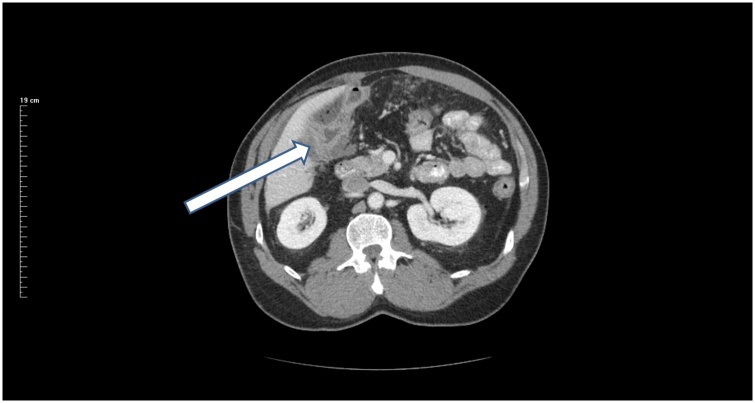
CT slice 4 months postoperatively through the upper abdomen. At the time of resection of recurrent cancer, tumor was located on the posterior surface of the liver. This anatomic site was a close margin of resection of the primary ascending colon cancer.

In June of 2018, the patient began noting problems with digestion and pain after eating. CEA had increased to 940 ng/ml. Repeat CT scan showed multiple nodules within the greater omentum compatible with peritoneal metastases. The liver, kidneys, and ureters were normal. There was marked stranding within the right colon resection site (Fig. 4). Bowel loops proximal to the prior ileocolic anastomotic site were moderately dilated and fluid-filled. A mass was noted in the abdominal wall at the laparoscopic port site. There was also a mass in the rectovesical space immediately adjacent or invading the right and left seminal vesicles.

In July of 2018, the patient underwent exploratory laparotomy. The posterior surface of the liver was layered by cancer. The undersurface of the right hemidiaphragm was covered by tumor nodules. The pelvis contained a 4 cm tumor mass invading into the seminal vesicles. The old ileocolic anastomosis was involved and needed to be resected. The umbilical port site was infiltrated by cancer. Near complete resection was possible by cytoreductive surgery.

Intraoperatively, the patient was treated with HIPEC-melphalan [11]. This postoperative course was unremarkable. The histopathology showed adenocarcinoma compatible with the primary tumor in all tissues submitted. The peritoneal cancer index was 28 and the CC score was 2 [12]. The resection was judged to be a palliative effort and its major goal was to relieve intestinal obstruction and to prepare the patient for rapid initiation of palliative systemic chemotherapy.

Neither of our patients was evaluated by the MDT preoperatively. Their preoperative and intraoperative management was evaluated in an attempt to improve the outcome of subsequent patients.

## 3 Discussion

### 3.1 Preoperative assessment was by CEA

Patient 1 had a preoperative CEA of 90 ng/ml and patient 2 had a preoperative CEA of 40.4 ng/ml. In the American Joint Committee on Cancer Prognostic Factors Consensus Conference, a preoperative CEA greater than 5 ng/ml was listed as a poor prognostic variable [13]. A high preoperative CEA indicated a guarded prognosis of our two patients who developed peritoneal metastases. The preoperative CEA should have been considered prior to primary cancer resection by the MDT.

### 3.2 Preoperative assessment by CT

A high quality CT of our patients was obtained preoperatively. It showed large masses in the region of the caecum (patient 1) and ascending colon (patient 2). Lymph node involvement in patient 1 was assessed and nodes were positive by size criteria (greater than 1.5 cm). In both patients, of special concern was the border of the primary malignancy. A real danger for narrow or positive margins of resection could have been recognized by a radiologist who was part of an MDT evaluation. Dighe and colleagues have shown that the borders of the primary cancer by CT have profound implications for the development of local recurrence and peritoneal metastases [14].

### 3.3 Documentation by the MDT regarding preoperative appreciation of the advanced stage of the primary tumor

The fair and proper assessment of a deficiency in the management of a primary colon or rectal cancer requires a direct link between deficient treatment and the adverse outcome. Was there treatment failure? Or was the adverse outcome unavoidable because the biology of the cancer was aggressive. To escape culpability an evaluation of all information available preoperatively must be documented. In neither of our patients was the marked CEA elevation and the concerning preoperative CT acknowledged. An absence of documentation in the preoperative written assessment of increased risk for inadequate containment with colon resection may indicate a management deficiency and a requirement for preoperative MDT evaluation.

### 3.4 Deficiencies in the resection technique

The surgeon attempted to resect, using routine colon resection technique, these large masses with positive radiologic margins. The resection in patient 1 was laparoscopic converted to open and in patient 2 laparoscopic. Both open or minimal access resections may result in spillage of tumor cells. In the resection of these two right colon cancers there may be links between the surgeon’s performance and the recurrent (progressive) disease that developed. At the time of the cytoreductive surgical procedures the sites of recurrent disease were documented intraoperatively and confirmed by the histopathology. In patient 1, the most prominent sites of disease progression were in the anatomic sites of narrow margins of resection. There was a large volume of adenocarcinoma at the right colon resection site and there were multiple cancer nodules along the course of the right ureter.

In patient 2, the posterior surface of the liver was a narrow margin and at reoperation this anatomic site was layered by cancer. The undersurface of the right hemidiaphragm had been seeded and was covered by countless tumor nodules. Drop metastases were in the pelvis. The old ileocolic anastomosis was involved and the umbilical port site infiltrated by cancer.

### 3.5 Advanced surgical treatment options for cancers at high risk

One may argue that there are no options other than a routine surgical technology by which to resect a right colon malignancy at high risk for tumor spillage by surgery. Or one may argue that the standard of care is routine colon resection followed by adjuvant systemic chemotherapy in all stage III patients. To the contrary, there are surgical treatment options that may help to avoid the spillage of cancer cells that will eventuate in local recurrence and peritoneal metastases. First, with a large primary tumor mass the length of an extraction site required for an atraumatic removal from the specimen from the peritoneal space is 12–15 cm. In this situation where a lengthy midline incision is required for specimen removal, a laparoscopic resection may offer no advantages over an open resection.

Second, a short course of FOLFOX or FOLFIRI neoadjuvant chemotherapy may shrink the large primary tumor and allow for more adequate margins of resection. A smaller size would reduce the possibilities for dissemination of cancer cells from surgical trauma. Randomized controlled trials are currently active to test this preoperative treatment strategy (FOXTROT) [15].

Third, as the large primary tumor is visualized either by laparoscopy or by laparotomy, its margin of resection must be assessed. If an infiltration of adjacent structures is identified with gentle movement of the primary cancer, plans to resect these structures en bloc along with the primary cancer should be formulated. Parietal peritonectomy procedures should be used to protect lateral and anterior margins [16]. Retroperitoneal fat along a right ureter stripped of surrounding soft tissue should be preserved on the primary cancer specimen. If, as in patient 1, preoperative CT suggests a posterior extension of the cancer with narrow margins on the right ureter, a preoperative right ureteral stent would be helpful to facilitate ureterolysis and an optimal margin along this structure. There should be not trauma to the primary cancer or to the adjacent ureter.

Finally, after the cancer and proximal and distal bowel have been removed, the specimen should be oriented and assessed by the pathologist. If the T stage is T_3_ or T_4_, hyperthermic intraperitoneal chemotherapy (HIPEC) or extensive intraperitoneal lavage (EIPL) should be used if available [[17], [18], [19]]. HIPEC or EIPL are theoretically of greater benefit if used prior to rather than after the anastomosis is constructed. If there are, despite maximal efforts to clear the primary cancer, positive margins, metal clips should be placed so that accurate postoperative radiotherapy may be considered by the multidisciplinary team.

### 3.6 Implications for changes in management of high risk colon cancer patients

Perhaps increased attention to preoperative evaluation of colon cancer patients by an MDT can result a time when the evaluation of cancer surgery will progress beyond adverse events and in-hospital or 60-day mortality. Currently, the assessments of the surgical events are short-term. As a matter of fact, these short-term assessments regarding morbidity and mortality can create a perverse incentive to minimize the extent of surgery. They may work against long-term outcomes in terms of treatment-related survival. The assessment of the adequacy of a colon cancer resection should include a long-term assessment of local and regional treatment failure, especially an assessment of local recurrence within the area of the cancer resection. It would also involve a longitudinal assessment of peritoneal dissemination of the cancer. Local recurrence and peritoneal metastases emanate from the same intraoperative spillage of cancer cells. This spillage occurs either prior to or at the time of the cancer resection. It is possible that patients may enter the operating room with a contained malignancy and leave with widespread dissemination within the peritoneal space.

### 3.7 Incidence of guarded prognosis patients to be evaluated by the MDT

The incidence of patients with primary colorectal cancer who can be identified to be in a poor prognostic group is difficult to establish because elevated CEA and concerning features on preoperative CT have not been included as criteria for a guarded prognosis. Of course, patients with perforated colon cancer or obstructed colon cancer are known to have a reduced prognosis. However, in these patients surgery to relieve their acute symptoms is required. The MDT does not have a window of opportunity to make recommendations regarding their preoperative management. There may be other poor prognostic indicators such as possible peritoneal metastases or limited liver metastases on preoperative CT scan. These patients with “oligometastases” also need MDT presentation prior to colon cancer resection. They may have treatment options other than simple colon cancer resection followed by systemic chemotherapy. A rough estimate would be that 1 in 10 patients with colon cancer can be identified as a patient with poor prognosis who also has the window of opportunity for preoperative treatments and special techniques intraoperatively in order to optimize the long-term outcome. All oncologists would agree that prevention of local recurrence and peritoneal dissemination is a much more effective management plan than treatment of these conditions after they have been diagnosed in follow-up at a later time.

Currently, when an oncologically inadequate resection of a primary cancer occurs, the patient may leave the hospital with no adverse events and an absence of mortality. He or she will usually be referred to a medical oncologist who will initiate systemic chemotherapy or radiation therapy. These referrals are seldom profitable and the removal of the primary malignancy is, unfortunately, for that patient only the beginning of a long fight against cancer. If local recurrence or peritoneal metastases develops in follow-up, inadequate management may have caused the death of the patient.

## Conflicts of interest

Paul H. Sugarbaker has no conflicts of interest to declare.

Mohamed T. Hassanein has no conflicts of interest to declare.

## Sources of funding

Data management and secretarial support provided by Foundation for Applied Research in Gastrointestinal Oncology.

## Ethical approval

Local IRB-approval for this case report was not required:

MedStar Health Institutional Review Board has determined that a case report of less than three (3) patients **does not meet the DHHS definition of research** (45 CFR 46.102(d)(pre-2018)/45 CFR 46.102(l)(1/19/2017)) **or the FDA definition of clinical investigation** (21 CFR 46.102(c)) and therefore are not subject to IRB review requirements and **do not require IRB approval**.

This case series is of 2 patients.

## Consent

Written and signed consent was obtained from the patients.

## Author’s contribution

Paul H. Sugarbaker, MD: study concept or design, data collection, data analysis or interpretation, writing the paper

Mohamed T. Hassanein, MD: study concept or design, data collection, data analysis or interpretation, writing the paper

## Registration of research

This study was registered as a case series on the www.researchregistry.com website with UIN 4417.

## Guarantor

Paul H. Sugarbaker, MD.

## Provenance and peer review

Not commissioned, externally peer-reviewed.

## CRediT authorship contribution statement

**Paul H. Sugarbaker:** Conceptualization, Data curation, Formal analysis, Funding acquisition, Investigation, Methodology, Project administration, Resources, Software, Supervision, Validation, Visualization, Writing - original draft, Writing - review & editing. **Mohamed T. Hassanein:** Conceptualization, Data curation, Formal analysis, Funding acquisition, Investigation, Methodology, Project administration, Resources, Software, Supervision, Validation, Visualization, Writing - original draft, Writing - review & editing.
